# Supplementary material for: Mouse MRI shows brain areas relatively larger in males emerge before those larger in females
Source: Nat Commun. 2018 Jul 5;9:2615. doi: 10.1038/s41467-018-04921-2 (PMC6033927; doi:10.1038/s41467-018-04921-2)
Supplement: Supplementary file 1 — Supplementary Information [file 41467_2018_4921_MOESM1_ESM.pdf]

Supplementary

Mouse MRI shows brain areas relatively larger in males emerge before those larger in females. Qiu *et al.*

Supplementary Methods

Behavioural assessment

To assess neonatal brain development and behavioural outcomes of mice undergoing scanning, developmental milestone and behavioural testing was conducted on additional groups of mice. Testing was conducted on three groups of mice. The first group consisted of scanned mice: these mice were scanned at postnatal days 3, 5 ,7, 10 and 17, and thus were exposed to isoflurane during the scans and to maternal $MnCl_2$ at postnatal days 2, 4, 6 and 9, as well as an intraperitoneal injection of $MnCl_2$ at postnatal day 16. The second group consisted of their non-scanned littermates. These mice were exposed to maternal $MnCl_2$ at postnatal days 2, 4, 6 and 9. The third group consisted of non-scanned control mice, who were not exposed to any isoflurane or $MnCl_2$. $MnCl_2$ administration and scanning procedures were described in the main text. Six cages of C57BL/6J mice were used, each culled to a litter size of 6, with the exception of one cage that had 5 pups. Four cages were randomly selected for scanning. 1-2 mice were used from each cage to be scanned, while the remainder of the mice were non-scanned littermates, resulting in 7 scanned mice (4 male, 3 female) and 17 non-scanned littermates (7 male, 10 female). There were 11 non-scanned control mice (4 male, 7 female).

Behavioural testing was done prior to $MnCl_2$ administration or scanning if both fell upon the same day. Righting reflex was conducted on postnatal days 4, 5, and 6. Prior to testing, the dam was removed from the home cage. Each pup was tested individually. The pup was placed on its back, and the time required for the pup to flip over and place all four paws on the ground surface was measured. If no righting took place within 30 seconds, the pup was picked up and righted manually. After all pups in one cage were tested, the dam was returned. Eye opening was observed daily from postnatal day 10 until day 17. Each mouse was given a score depending on number of eyes open: 0 for no eyes open, 1 for one eye open and 2 for both eyes open. Open field was conducted on postnatal day 16. Behaviour was recorded in an arena that was 44 cm^2, with 16 beams on each axis (1 inch apart) (Med Associates Inc, Fairfax VT). Each arena was enclosed inside a sound attenuating chamber. The light intensity used was 200 LUX in the centre, and 150 LUX in the periphery. Data was collected with Activity Monitor 7 (Med Associates Inc, Fairfax VT). Six mice were tested at a time. Length of neonatal open field testing was 30 minutes. Time spent in the centre of the open field, as well as total ambulatory distance were measured. Following testing, mice were returned to their home cage. Open field was performed for a second time at postnatal day 65. The length of testing was 10 minutes. For an unrelated reason, one of our cages was terminated prematurely by veterinarian technicians, thereby decreasing our number of female scanned mice by one, and our female non-scanned littermates by two.

Righting reflex time and eye opening score were compared across scanned (S), non-scanned littermates (L) and non-scanned control (C) animals by running three linear mixed-effects models, and then comparing across models to assess whether group or sex-group interaction had a significant effect. The first linear mixed-effects model had fixed effects of group, postnatal day, sex, and their interactions, with a random effect of individual mouse. The second model had fixed effects of postnatal day, sex, and their interaction, and a random effect of individual mouse. These two models

were compared with a likelihood ratio test to assess whether group affected righting reflex time or eye opening score. A third model was run with fixed effects of group, postnatal day, group-postnatal day interaction, sex and a sex-postnatal day interaction, with a random effect of individual mouse. This third model was compared to the first model with a likelihood ratio test to assess whether there was a group by sex interaction. For open field, time spent in the centre of the open field and total ambulatory distance were analysed using linear models.

Registration

Affine and Non-affine Registrations
We illustrated the registration procedure in Supplementary Figure 11 (top row), which shows the registration of the p3 average (source image) to the p5 average (target image). The registration procedure was composed of two stages: affine registration performed using the `mni_autoreg` tools [1] and non-affine registration performed using the ANTs toolkit [2]. The two images were overlaid (middle image, first row), demonstrating that the source image needed to be distorted to fit the target image. After the affine registration was performed, the resultant transformation was used to transform the source image and overlay it on the target image (middle image, second row). It was clear that the alignment has improved but was still unsatisfactory in some brain regions (third row). After the non-affine registration was performed, the source image was transformed using both the affine transformation and the non-affine transformation. This procedure resulted in satisfactory alignment between the two images (fourth and fifth row).

Jacobian Determinants
Jacobian determinants were used to quantify the volumetric changes caused by deformations. In Supplementary Figure 12, we illustrated the concept of absolute Jacobian determinants. Gridlines in the target image cerebellum were warped upon transformation to the source image. The determinants of this transformation are called the absolute Jacobian determinants. It was computed for every voxel and the resulting voxel map was overlaid on the target image. This illustrated how absolute Jacobian determinants capture the extent to which regions in the source image were smaller or larger than the corresponding regions in the target image. A similar procedure was applied to find relative Jacobian determinants. Gridlines in the target image cerebellum were warped upon transformation to the affine-transformed source image. The determinants of this transformation are called relative Jacobian determinants and its voxel map was overlaid on the target image. Relative Jacobian determinants captured volumetric changes after scaling brains to the same size.

Generating Atlas Labels

To test for biases, we compared three different sets of atlases (illustrated by Supplementary Figure 13). The first is called a consensus atlas and was used throughout the main study (detailed in Methods). In the second atlas set (called the resampled atlases), we resampled the consensus atlas (atlas on the p65 average) to each of the other 8 time points using the Level 2 transformations and nearest-neighbour interpolation. This atlas specifically removes biases in structure volumes

associated with resampling done in the Level 2 registration. However, it does not remove biases associated with using a single atlas as the starting point for structure analysis.

The bias associated with using a single atlas can only be removed by manual segmentation of age-consensus averages. Since this process is quite intensive, we instead chose to use the MAGeT [3] pipeline to generate atlases for each age from multiple intermediate atlases. First, the p65 atlas and its associated MRI average were transformed to each individual p65 image using the transforms obtained from the p65 Level 1 registration. Each of the individual p65 resampled atlases were then used as starting atlases in the MAGeT pipeline to segment the average from the earlier adjacent time point: p36 average. In this pipeline, each atlas was registered to the p36 average, followed by a voxel-voting step to determine the label given to each voxel. At the end of these steps, the p36 average had an atlas overlaid on it, and this atlas never used the information that came from Level 2 of our registration. The exact procedure was then applied to the p36 atlas to generate the p29 atlas, and then the p29 atlas was used to generate the p23 atlas, and so on until all the time points had atlases associated with them. While a p65 atlas began this procedure, Level 2 registration information was never used, multiple atlases were generated in intermediate steps, and each time point's atlas only depended on the atlas of the time point immediately older. While these reasons do not eliminate longitudinal registration bias in this third atlas set (called the voted atlases), they greatly reduce it.

Gene Expression

The underlying gene expression changes associated with sexually dimorphic neuroanatomy (Figure 6) remains unknown. We wanted to identify candidate genes that might be associated with these dimorphisms. To do so, we used the genome-wide spatial gene expression data available from the Allen Brain Institute [4] and compared them to our neuroanatomical results. There are, however, four caveats associated with using gene expression data for our purpose.

The first caveat is that genome-wide gene expression data was only collected in males at p56. While some genes have developmental gene expression at p4, p14, and p28 [5], most genes do not and there is no gene expression data for females. The second caveat is that most genes had their expression data come from only one mouse. The third caveat is that the majority of gene expression data was collected using ISH (*In situ* hybridization) on sagittal slices spanning only one hemisphere. Only a small subset of genes had ISH conducted on coronal slices spanning the whole brain. Lastly, despite extensive quality-control steps taken by the Allen Brain Institute, several regions in the brain were missing gene expression data. To compensate for this, whenever any gene had multiple replicates, we chose the replicate with the least amount of missing data. Furthermore, we excluded experiments where expression data spanned less than 20% of the brain. While these caveats do limit the conclusions made from this analysis, spatial gene expression data is still useful in identifying candidate genes associated with sexual dimorphisms for further exploration.

Neuroanatomy Prediction

We wanted to predict the absolute volumes of structures. To model this growth, we used natural spline functions. Nth-order natural splines are characterized by N basis functions of age t, where the kth basis function is represented by $f_k(t)$. For each structure, the structure volume y_{ij} in the training data was fit with the following model:

$$y_{ij} = \alpha_1 + \alpha_2 s_i + \sum_{k=1}^{N} \alpha_{k+2} f_k(t_{ij}) + \sum_{k=1}^{N} s_i \alpha_{k+N+2} f_k(t_{ij}) + \beta_{0i} + \varepsilon_{ij} \tag{12}$$

Variables in this model are described in the main text. While increasing the order N of the natural splines allows one to model more complex growth curves, it can also overfit data leading to inaccurate predictions for data outside the training set. We computed Bayes Factors, using the `BayesFactor` package [6], to decide which order N of natural splines to use. Bayes Factors compute the evidence any model (12) has versus an intercept only model ($y_{ij} = \alpha_1 + \varepsilon_{ij}$) and assumes a reasonable set of priors on the predictors. We used the `BayesFactor` package's default Jeffreys prior for our analysis. By finding the Bayes Factor associated with Model (12) for values of spline order N ranging from one (linear growth) to the number of time points in the data minus one. The spline order N chosen for the structure's model is the one with the highest Bayes Factor associated with it.

Once we determined the order N of the natural splines modelling fixed growth effects, we wanted to similarly model random growth effects with Mth-order natural splines and optimize M. The training data was fit with the model below:

$$y_{ij} = \alpha_1 + \alpha_2 s_i + \sum_{k=1}^{N} \alpha_{k+2} f_k(t_{ij}) + \sum_{k=1}^{N} s_i \alpha_{k+N+2} f_k(t_{ij}) + \beta_{0i} + \sum_{k=1}^{M} \beta_{ki} f_k(t_{ij}) + \varepsilon_{ij} \tag{13}$$

After fitting multiple models with different values for M, we chose the model with the lowest Bayesian information criterion (BIC) [7] to predict structure volumes.

Lastly, we also placed a gaussian weighting on data in the training set depending on the age. This step was motivated by the fact that when predicting volumes at a particular age, the time point closest to that age is the most informative. However, only considering the closest time point alone may be less informative than taking some information from the other time points. To balance the two extremes, we placed a gaussian weighting on the data. The gaussian weighting was centered on the time t which we want to predict and its spread parameter (σ^2) can be optimized. A high σ^2 implies data over all time is weighted equally by the model, and a low σ^2 implies data closest to the prediction time t is weighted higher than data further away. This spread parameter was optimized using leave-one-out cross validation.

The model described above was used primarily in our study. However, we also explored two improvements to our model to check for consistency. The first was adding a covariate for total brain volume $V_{i,j}$ at the time point prior to the one being predicted ($j \to j-1$), which was done to control for whole-brain volume effects in subjects. The model with this covariate is given below, and fixed

N splines and random M splines are optimized as detailed above:

$$y_{ij} = \alpha_1 + \alpha_2 s_i + \sum_{k=1}^{N} \alpha_{k+2} f_k(t_{ij}) + \sum_{k=1}^{N} s_i \alpha_{k+N+2} f_k(t_{ij}) + \alpha_{2N+3} V_{i,(j-1)} + \beta_{0i} + \sum_{k=1}^{M} \beta_{ki} f_k(t_{ij}) + \varepsilon_{ij} \quad (14)$$

The second improvement was to use a random forest. Thus far, structures were modelled independently of each other, i.e. a structure's volume at a certain time t was predicted from the same structure's volume at earlier times. Using the random forest machine learning method, we can predict a structure's volume from other structures at an earlier time. To do so, we first fit the primary model described above to the training data and obtained the residuals of this model at the age t we wanted to predict. We then identified the volume of all 182 structures in the brain at the immediate earlier time and used them to model these residuals using a random forest from the `randomForest` package [8]. The random forest contained 500 trees and randomly sampled 5 structures at each tree split. The final predicted value was the sum of predicted values from both the initial model and the random forest.

Optimizing Growth Models for Absolute Determinants

Absolute volumes required more complex growth curves than relative volumes. To model this curve, we used a similar procedure as in the previous section. We found the Bayes Factor associated with Model (12) for values of spline order N (fixed effect of growth) ranging from 1 to 8. For 80% of structures, Bayes Factor was maximized by splines of order $N \leq 6$. The data was then fit with the model defined by Model (13) with $N = 6$ and the optimized M (spline order associated with random effect growth) is determined by finding the model with the minimum BIC. We found that 95% of structures were best fit by $M \leq 2$. Thus, we chose order-6 natural splines for fixed effects of age and order-2 natural splines for random effects of age to fit absolute Jacobian determinants. We computed the likelihood-ratio statistic comparing this optimized model to a similar one without sex and sex-age interactions to ascertain significance of sex on absolute determinants.

Supplementary Discussion

Registration Bias

The first level in our longitudinal registration consists of nine independent registrations, each of which registers all the scans from one time point to an age-consensus average. As such, nine age-consensus averages, one for each age, are created from the first level registration. In the second level, each time point's age-consensus average is registered to the age-consensus average of the immediate next time point, with the exception for p65—the final time point. We chose to make the p65 age-consensus average the registration consensus average, i.e. the common space to which all subjects and time points are compared to for statistical analysis. While any time point can be chosen for analysis, we picked this age as it is close to the MRI atlas (p60) and the Allen Brain Gene Expression atlas (p56). While it is a necessary part of our analysis, it is important to note that the practice of picking a consensus time point can lead to biases. For example, interpolation bias may be a factor as, one time point (p65 in our case) receives less interpolation than the other time points. Furthermore, these biases may not affect all groups equally [9]. Below, we show that our registration is not biased across sex or individuals, and that the statistics maps generated in our study are similar regardless of the time point chosen as the consensus time point.

Interpolation bias has little effect on voxelwise statistics
We regenerated our statistics map (Figure 4) after picking p17 as the registration consensus average (Supplementary Figure 9). This time point was chosen as it was the median time point in our study, which follows more closely to literature recommendations [9]. This map was then resampled to p65 space to facilitate comparison of the two statistics maps. The high similarity of Figure 4 and Supplementary Figure 9 show that the effect of choosing p65 or the p17 median time point on sexual dimorphisms detected is small.

Next, we tested whether detection of sexual dimorphisms at any age would be influenced by picking the p65 age-average as the registration consensus average. The two-level registration generates two sets of determinants: one set from Level 1 and one from Level 2. In our main study, we use the determinants from Level 2 as these are all transformed to a consensus p65 space. The determinants of each of the Level 1 registrations are in the space of their respective age-consensus average. For example, Level 1 determinants from the p17 registration are registered to the p17 consensus average and Level 2 determinants from the p17 registration are registered to the p65 consensus average. Note that longitudinal registration bias would only exist in the Level 2 determinants and not the Level 1 determinants as the nine Level 1 registrations (one for each time point) occurred independently and are agnostic to each other. We wanted to test whether statistics determined from Level 1 determinants are similar to statistics generated from Level 2 determinants.

At every time point, we first computed the effect size (9) statistic at every voxel associated with log absolute determinants from Level 1 comparing male and female mice. Similarly, at every time point, we then computed the effect size for the log absolute determinants generated in Level 2. These Level 2 effect size statistics were then transformed to their corresponding Level 1 age-consensus average and the correlation between the Level 1 effect size map and the transformed Level

2 effect size map was computed. For example, we took the p17 male and female log determinants from Level 1 and computed a voxelwise effect size map. This map was registered to the p17 consensus average as determined by the p17 Level 1 registration. We then took the p17 male and female log determinants from Level 2 and computed a voxelwise effect size map. This map was registered to the p65 consensus average and was then transformed to the p17 consensus average. The voxelwise correlation between the Level 1 and transformed Level 2 effect size maps was then used to test if using Level 2 determinants (which are results of the longitudinal registration) incurred significant bias when compared to Level 1 determinants (which are agnostic to the longitudinal aspects of the data). We found that the Level 1 and transformed Level 2 determinants correlated very strongly (dotted vertical line in Supplementary Figure 10). Furthermore, instead of computing effect sizes associated with sex, we repeated the above procedure with arbitrary spatial statistics patterns, which we computed by calculating effect sizes after random permutations of the sex label. The correlations between Level 1 and transformed Level 2 spatial patterns were also quite high and reported as a density plot (Supplementary Figure 10). Taken together, this indicated that the biases in the statistics maps generated from our longitudinal registration (whether they are associated with sex or not) are minimal.

Atlas-based bias does not discriminate individuals or sex

We compared structure volumes for every subject and at every time point using each of the three sets of atlases: the atlas placed on the p65 brain (called the consensus atlas), the resampled atlases from each age, and the voted atlases from each age. Supplementary Figure 14A shows the volume of the Lobule 1-2 white matter located in the cerebellum. While the consensus and resampled atlases were slightly different from each other, the voted atlas was very different from them both. In fact, according to the voted atlas, the structure does not exist before 5 days of age. This demonstrates that the particular atlas chosen does play a role in the volumes calculated. However, we sought to test specifically if this effect would apply differently across individuals or sex. To do so, we computed the z-score of the structure—i.e. subtracted the mean volume at every age and scaled by the volume standard deviation at every age (Supplementary Figure 14B). We observed that the atlas method did not play a significant role in affecting volumes of individuals or sex. This was tested using 3 linear mixed-effects models. The first model had z-score volumes as a response variable; fixed effects of time point, sex, and atlas (Consensus, Resampled, or Voted), as well as all interactions; and random effect of individual and individual-atlas interaction. The second model had the same fixed and random effects, but without the individual-atlas interaction. The third model was similar to the first but lacked the sex-atlas interaction fixed effect. The log-likelihood ratio test comparing the first and second model ascertains whether the atlas method had dissimilar bias across individuals, and comparing the first and third models ascertains dissimilar bias across sex. For the Lobule 1-2 white matter (Figure 14B), we found that the bias from using different atlases on z-score structure volume did not differ strongly across individuals or sex. We repeated this process for all structures in the mouse brain and found similar results as log-likelihood test showed no structures had uncorrected p-values below 0.90. This indicates that while the atlas used plays a role in the measurement of structure volumes through time, this bias does not apply differently to certain individuals or sexes.

Cortical Thickness and volume measurement agree on areas larger areas in females emerging in post-pubertal life

Regions larger in males emerge in early life and regions larger in females emerge in later life. Supplementary Figures 2–4 show that this pattern is consistent across different canonical measures of neuroanatomy. For both relative and absolute volumes, we identified the top 5% of voxels largest in males and top 5% of voxels largest in females at p65. Similarly, for absolute cortical thickness, we identified the top 5% of vertices thickest in males and top 5% thickest vertices in females at p65. This was done to ensure a consistent size of voxel or vertex elements for clustering. We then clustered these voxels and vertices by their effect size trajectories over time and identified three clusters. The pattern of larger/thicker areas emerging in early life in males and later life in females held across the different measures of neuroanatomy.

Additional Models for Sexually Dimorphic Development

Since the last point (p65) was almost a month after the second-last time point (p36), we repeated our analysis excluding the last time point and found similar regions exhibiting sexual dimorphisms (Supplementary Figure 16) compared to Figure 4. The model used for identifying sexually dimorphic regions, Equation (4), was modified to use time point τ_k (where k goes from 1 to 9 for each of our experimental time points) instead of age as a predictor. The results (Supplementary Figure 15), however, were still similar to Figure 4. Finally, we also found consistency with absolute volumes by computing a similar statistic for absolute log determinants (Supplementary Figure 17) after optimizing for the best growth model.

Supplementary Tab. 1: Variance of Sexually Dimorphic Structures in Males and Females

$10^3\times$ Variance of Absolute Volume (mm^3)								
	Bed Nucleus of The Stria Terminalis		Medial Preoptic Nucleus		Medial Amygdala		Periaqueductal Gray	
Age	M	F	M	F	M	F	M	F
3	1.2200	3.1700	0.0147	0.0644	1.0200	2.5200	18.4000	45.4000
5	2.5000	2.5300	0.0377	0.0597	1.6800	2.0400	27.3000	43.8000
7	1.6900	1.5700	0.0266	0.0277	1.2400	1.4000	19.3000	13.3000
10	1.7600	2.6800	0.0442	0.0511	1.4300	1.6800	25.8000	20.4000
17	1.1000	1.0700	0.0227	0.0327	1.0500	1.3100	16.3000	7.9100
23	2.9800	0.7730	0.0332	0.0271	2.2700	1.4300	28.2000	6.0400
29	0.8970	1.0200	0.0321	0.0233	0.7980	0.9380	16.7000	4.7200
36	1.2300	0.9280	0.0153	0.0260	0.8970	1.0700	11.1000	8.4800
65	1.0500	0.6390	0.0248	0.0289	2.0600	0.9680	13.2000	3.9900

$10^9\times$ Variance of Relative Volume (% Brain)								
	Bed Nucleus of The Stria Terminalis		Medial Preoptic Nucleus		Medial Amygdala		Periaqueductal Gray	
Age	M	F	M	F	M	F	M	F
3	299	774	19.2	46.9	234	452	6170	19700
5	399	1070	31.9	24.2	359	306	10100	10500
7	568	339	22.8	15.9	271	354	8870	6930
10	318	284	17	10.5	142	249	1810	3570
17	693	343	13.7	8.65	319	307	2020	2860
23	345	228	7.61	6.66	470	223	5410	7290
29	328	429	6.73	8.01	268	372	5500	2550
36	165	266	6.62	15.4	273	270	4990	4190
65	174	142	15.3	8.42	705	112	3400	2700

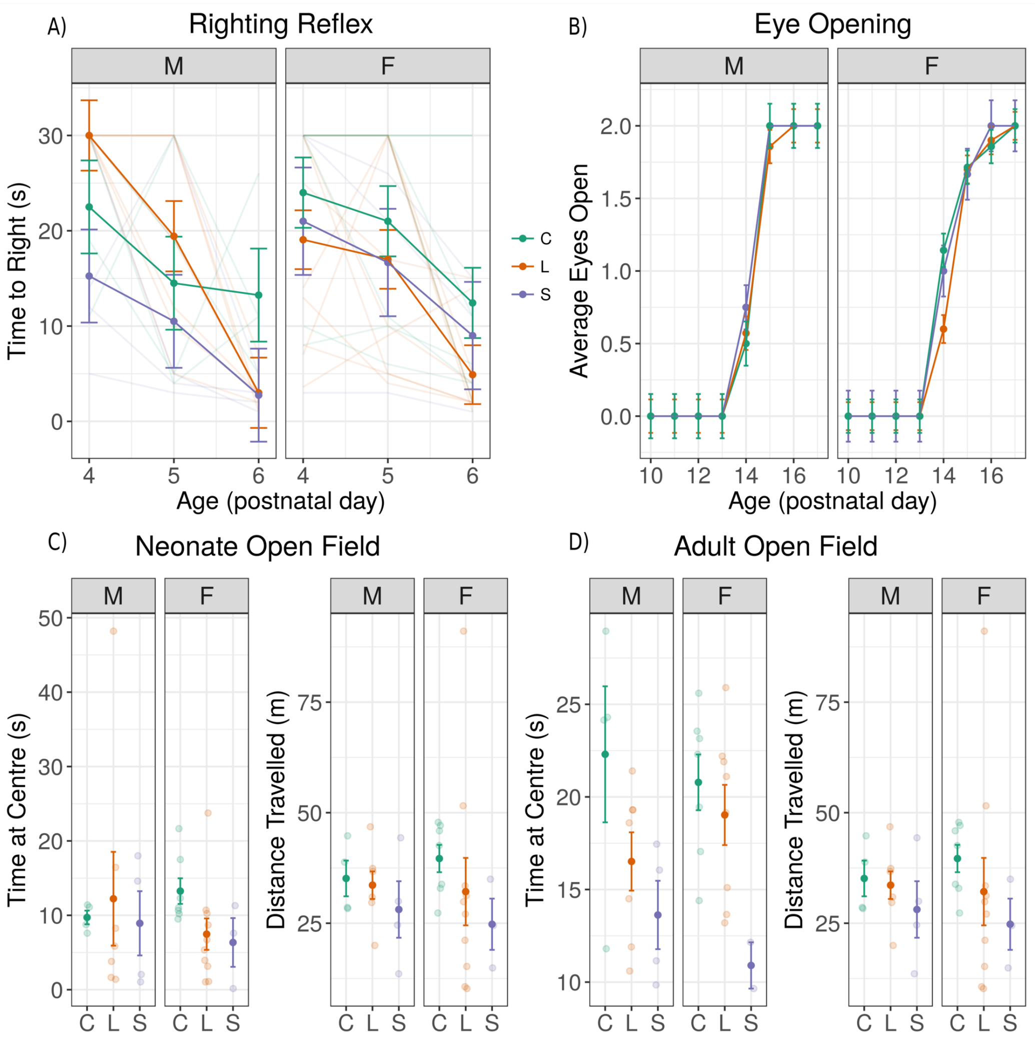

Supplementary Fig. 1: Time to right, eye opening, as well as time spent in centre and total ambulatory distance travelled in the open field was assessed neonatally across scanned mice (S), their non-scanned littermates (L) and non-scanned controls (C). A) There was no effect of group ($\chi^2_8 = 14.54$,P= 0.07), nor was there a group-sex interaction ($\chi^2_4 = 6.59$,P= 0.16) on time it took for pups to right themselves across postnatal days 4, 5 and 6. B) There was also no effect of group ($\chi^2_{32} = 20.61$,P= 0.94) or a group-sex interaction effect ($\chi^2_{16} = 9.30$,P= 0.90) on when eyes opened across postnatal days 10 to 17. For both A and B, linear mixed-effects models were used to create trendlines and bars representing standard error. C) There was no effect of group ($F_{2,29} = 0.47$,P= 0.63) or a group-sex interaction ($F_{2,29} = 0.64$,P= 0.54) on time spent in the centre of the open field at postnatal day 16, nor was there an effect of group on total ambulatory distance travelled ($F_{2,29} = 1.16$,P= 0.33) or a group-sex interaction ($F_{2,29} = 0.17$,P= 0.85). Thus, no neonatal behavioural metrics collected were significantly impacted by scanning, and the results were the same across both males and females. These mice were kept for further testing in the open field as adults (postnatal day 65). Although there was no group-sex interaction on centre time ($F_{2,26} = 0.91$,P= 0.41), there was a significant effect of group ($F_{2,26} = 6.81$,P= 0.004) as scanned mice spent less time in the centre of the open field compared to nonscanned controls (post hoc Tukey test $P_{adj} = 0.003$). Total ambulatory distance, however, did not show any significant differences across group ($F_{2,26} = 1.37$,P= 0.27), or by sex within each group ($F_{2,26} = 1.34$,P= 0.28). For both C and D, mean and standard error (bars) were calculated using linear models.

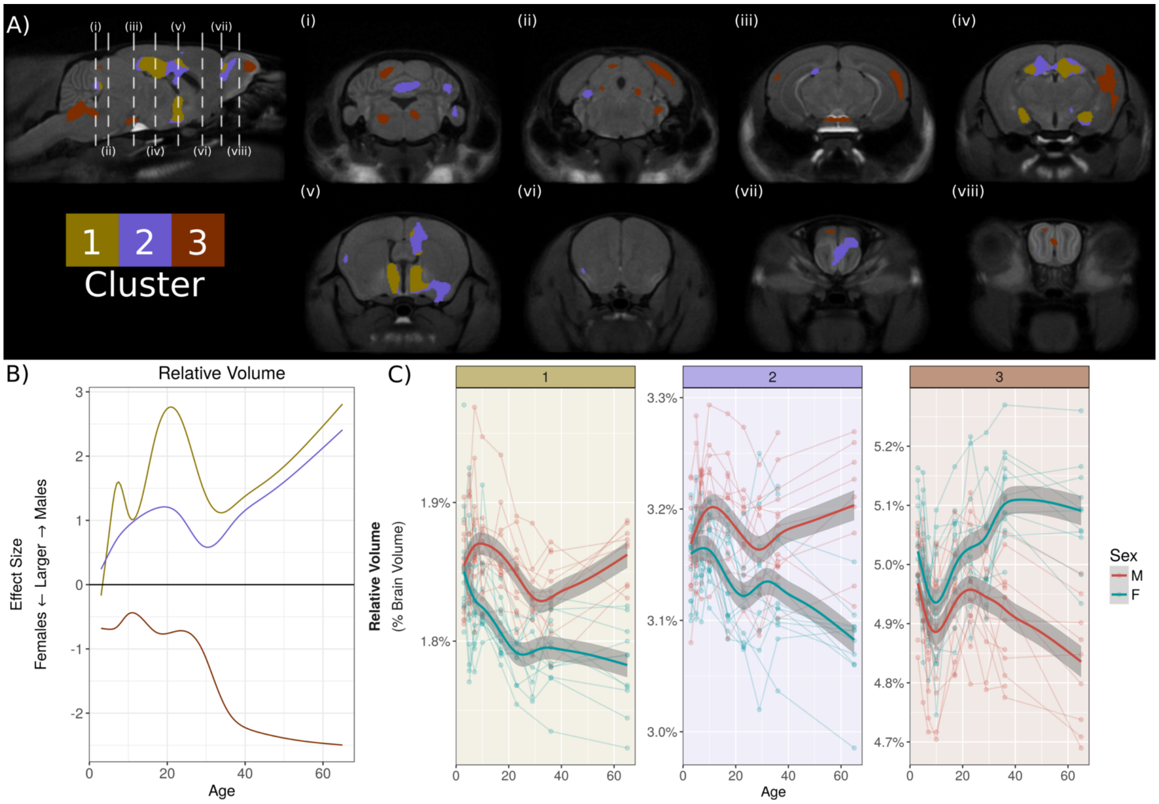

Supplementary Fig. 2: Top 5% of largest voxels (relative to whole brain) in males and females clustered by their effect sizes over time. Cluster 1 and 2 correspond to regions larger in males in adulthood and these sexual dimorphisms emerge early. Cluster 3 corresponds to regions larger in females and emerges around puberty.

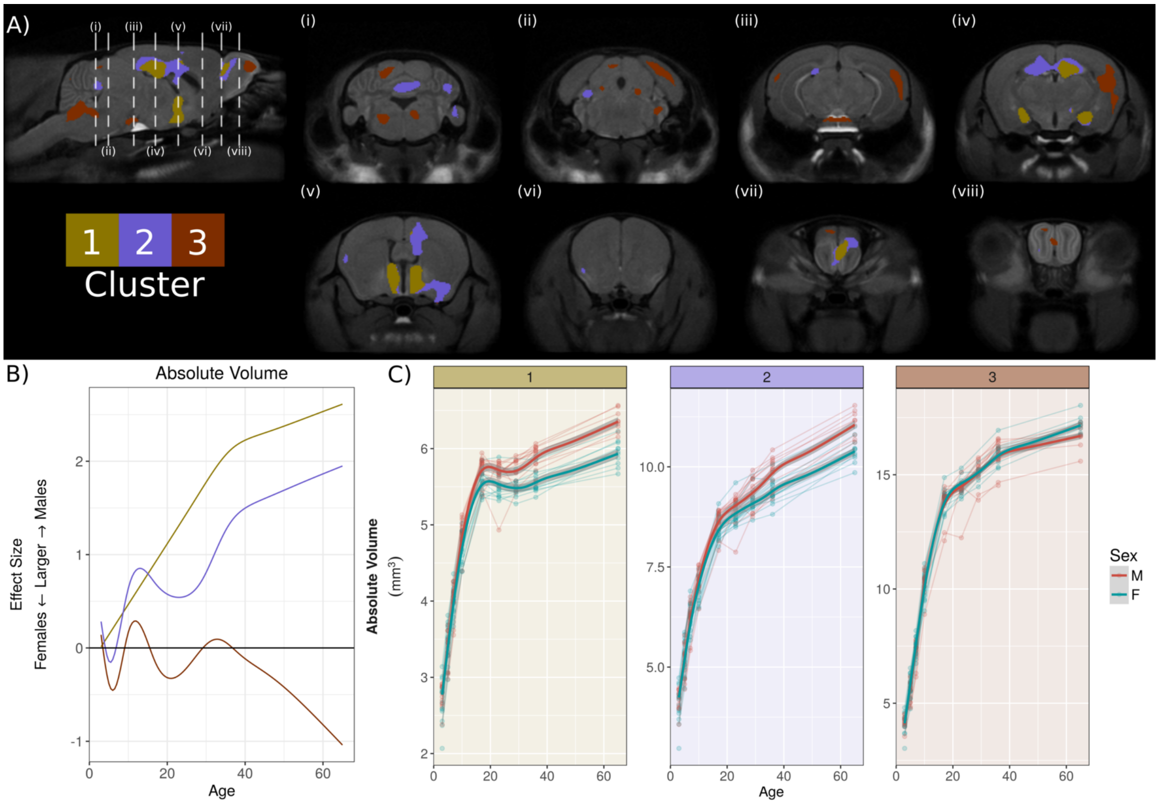

Supplementary Fig. 3: Top 5% of largest voxels in males and females clustered by their effect sizes over time. Similar to the case with relative volumes, Clusters 1 and 2 correspond to regions larger in males in adulthood and these sexual dimorphisms emerge early. Cluster 3 corresponds to regions larger in females and emerges around puberty.

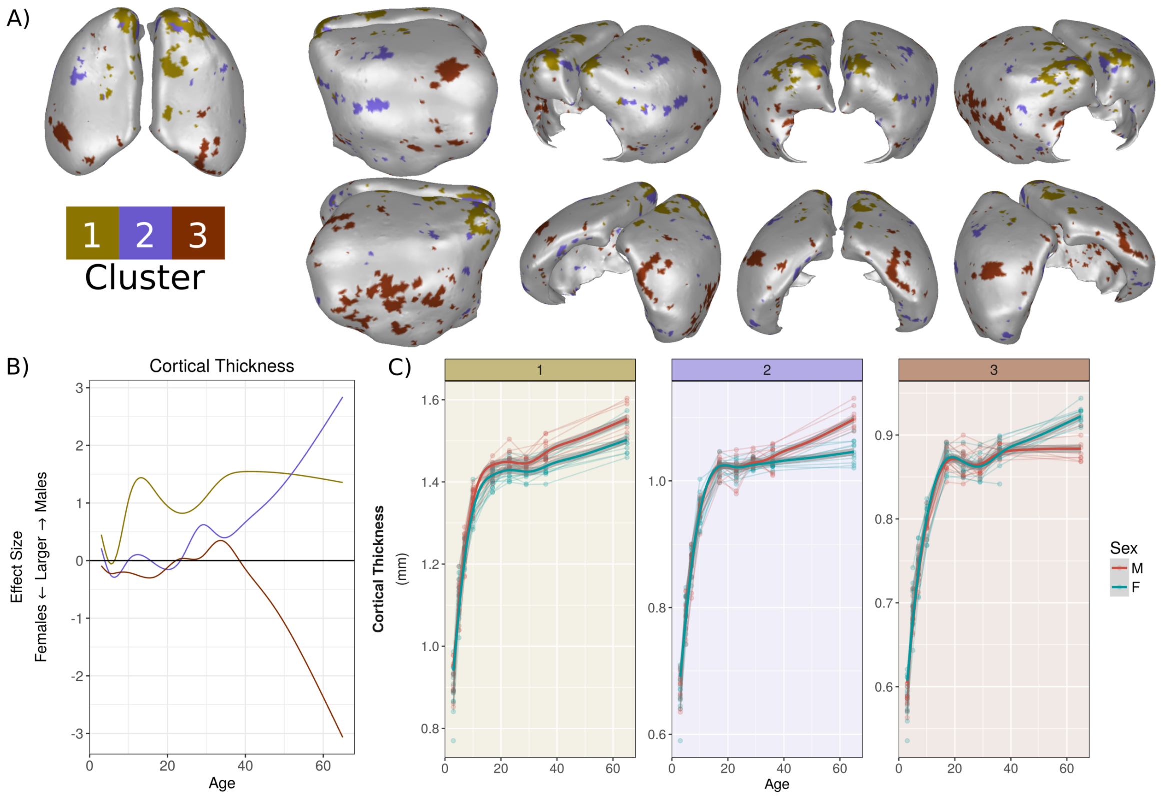

Supplementary Fig. 4: Top 5% of largest vertices in males and females clustered by their effect sizes over time. Similar to relative and absolute volumes, Clusters 1 corresponds to regions larger in adult males and these dimorphisms occur early in development, while Cluster 3 corresponds to regions larger in females and these dimorphisms occur around puberty. Similarly, Cluster 2 in both the thickness analysis and volume analysis corresponds to regions larger in males whose dimorphisms emerge after the regions in Cluster 1. However, while volume analysis had both Cluster 1 and Cluster 2 dimorphisms emerging in the first 10 days of life, cortical thickness analysis shows dimorphisms in Cluster 2 emerging around male puberty.

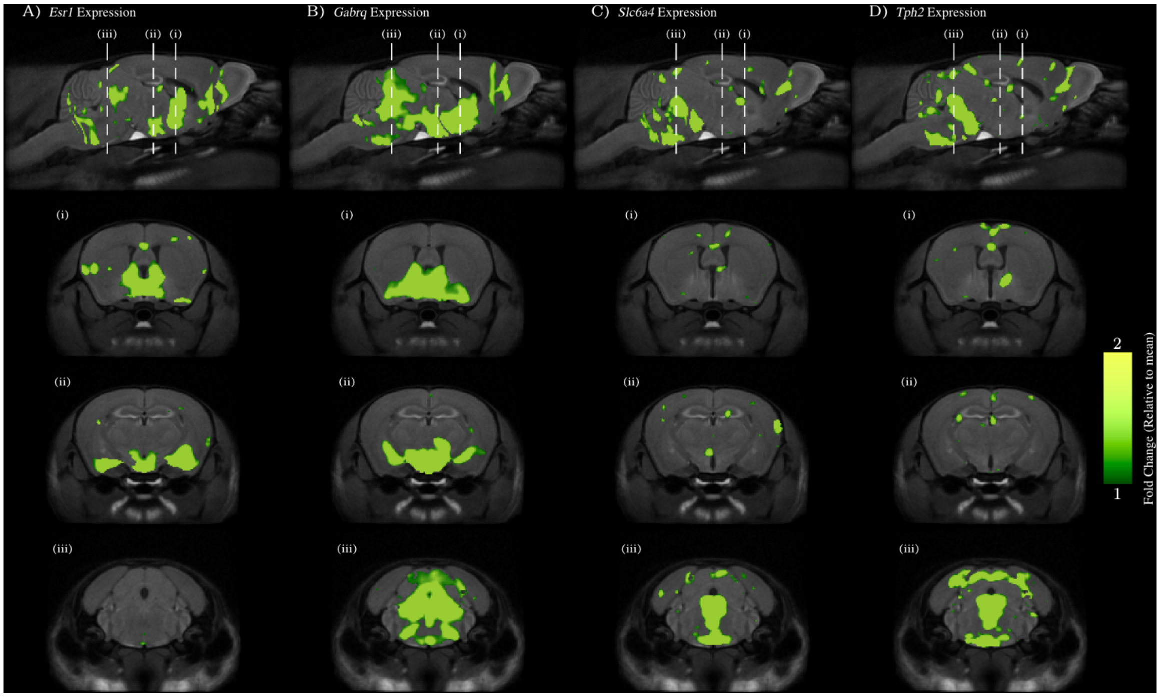

Supplementary Fig. 5: Preferential spatial expression of genes involved with sex processes in sexually dimorphic regions. A) Estrogen Receptor 1 (*Esr*1) has biased expression in BNST, MPON, and MeA; and its expression is 2.1 times higher in sexually dimorphic regions than its average gene expression in the brain. B) GABA A receptor, subunit theta (*Gabrq*) has biased expression in BNST, MPON, and MeA (regions larger in males), as well as the midbrain and hindbrain (regions larger in females) with a fold change 1.94 relative to mean. C) Solute Carrier Family 6 (Neurotransmitter Transporter, Serotonin), Member 4 (*Slc6a4*) had the highest preferential expression of any gene measured (fold change 3.7) and D) Tryptophan hydroxylase 2 (*Tph2*) had the second highest preferential expression (fold change 3.4).

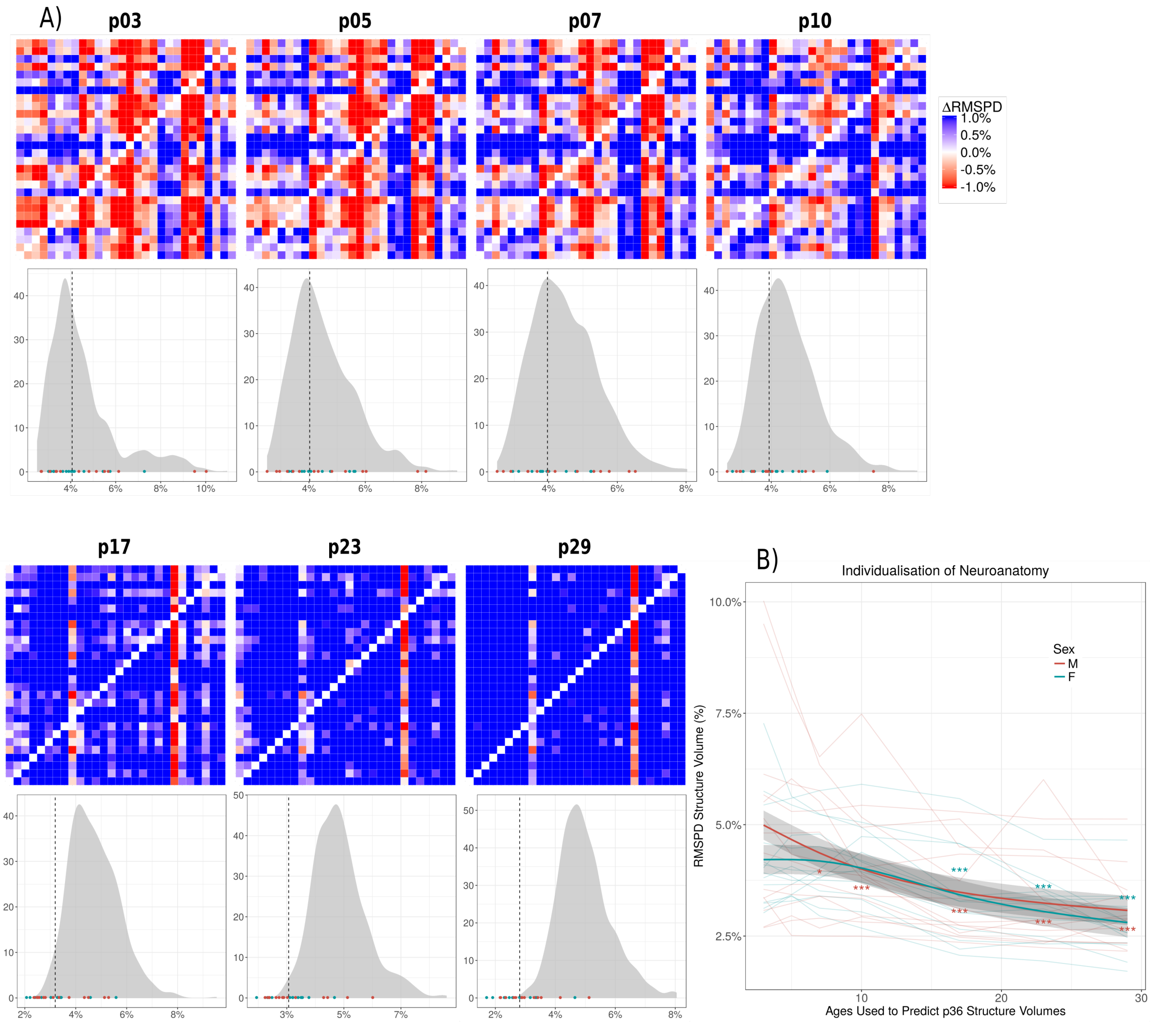

Supplementary Fig. 6: Using RMSPD to measure accuracy shows a similar pattern to RMSD as seen in Figure 8. A) Matrices show RMSPD values between each set of predicted structure volumes (columns, 1 per subject) and observed structure volumes (rows, 1 per subject), shifted such that the diagonal (prediction and observation RMSPD for the same subject) is 0. Red off-diagonals indicate that predictions for Subject X match observations for another subject better than observations for Subject X. The more red off-diagonal terms, the less specific the predictions are. Blue off-diagonals indicate specific predictions for Subject X as it matches observations of Subject X better than other subjects. Off-diagonal RMSPD are shown in a density plot (grey) and the diagonal RMSPD are given by points on the same plot (median is the vertical line). As data from further in development is included, model predictions become more specific. For example, model prediction specificity for p3 (which only uses data from Subject X at p3 to make predictions for Subject X at p36) is poor (58% chance of off-diagonal terms being blue) and prediction specificity for p10 (which uses data from Subject X at p3, p5, p7, and p10 to make predictions for Subject X at p36) is much better (75% chance of blue off-diagonal terms). B) RMSPD decreases (accuracy increases) as subject data from later in development are used in prediction. Male accuracy improves earlier than female accuracy.

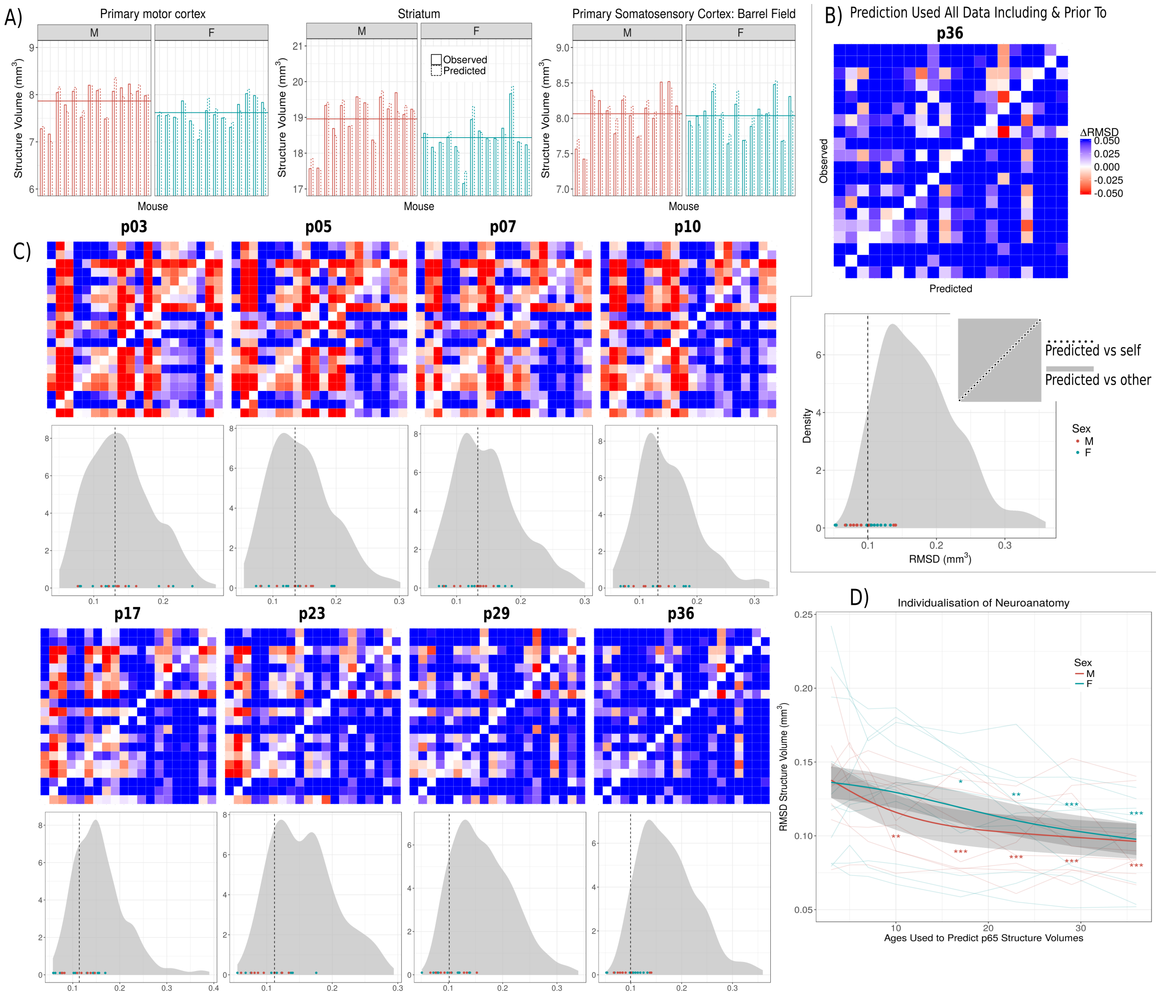

Supplementary Fig. 7: Predicting p65 structure volumes showed similar patterns of individualisation as predicting p36 volumes (Figure 8). A) Observed and predicted structure volumes for three representative structures with averages for each sex given by horizontal lines. Prediction for subject X at p65 was trained on all data excluding Subject X at p65. Model does not memorize the average, but instead fits individualised patterns in neuroanatomy B) Matrix shows RMSD values between each set of predicted structure volumes (columns, 1 per subject) and observed structure volumes (rows, 1 per subject), shifted such that the diagonal (prediction and observation RMSD for the same subject) is 0. Red off-diagonals indicate predictions for Subject X match observations for another subject better than observations for Subject X. The more red off-diagonal terms, the less specific the predictions are. Blue off-diagonals indicate specific predictions for Subject X as it matches observations of Subject X better than other subjects. Off-diagonal RMSD are shown in a density plot (grey) and the diagonal RMSD are given by points on the same plot (median is the vertical line). C) As data from further in development is included, model predictions become more specific. For example, model prediction specificity for p3 (which only uses data from Subject X at p3 to make predictions for Subject X at p65) is poor (53% chance of off-diagonal terms being blue) and prediction specificity for p17 (which uses data from Subject X at p3, p5, p7, p10, and p17 to make predictions for Subject X at p65) is much better (76% chance of blue off-diagonal terms). D) RMSD decreases (accuracy increases) as subject data from later in development is used in prediction. Male accuracy improves earlier than female accuracy.

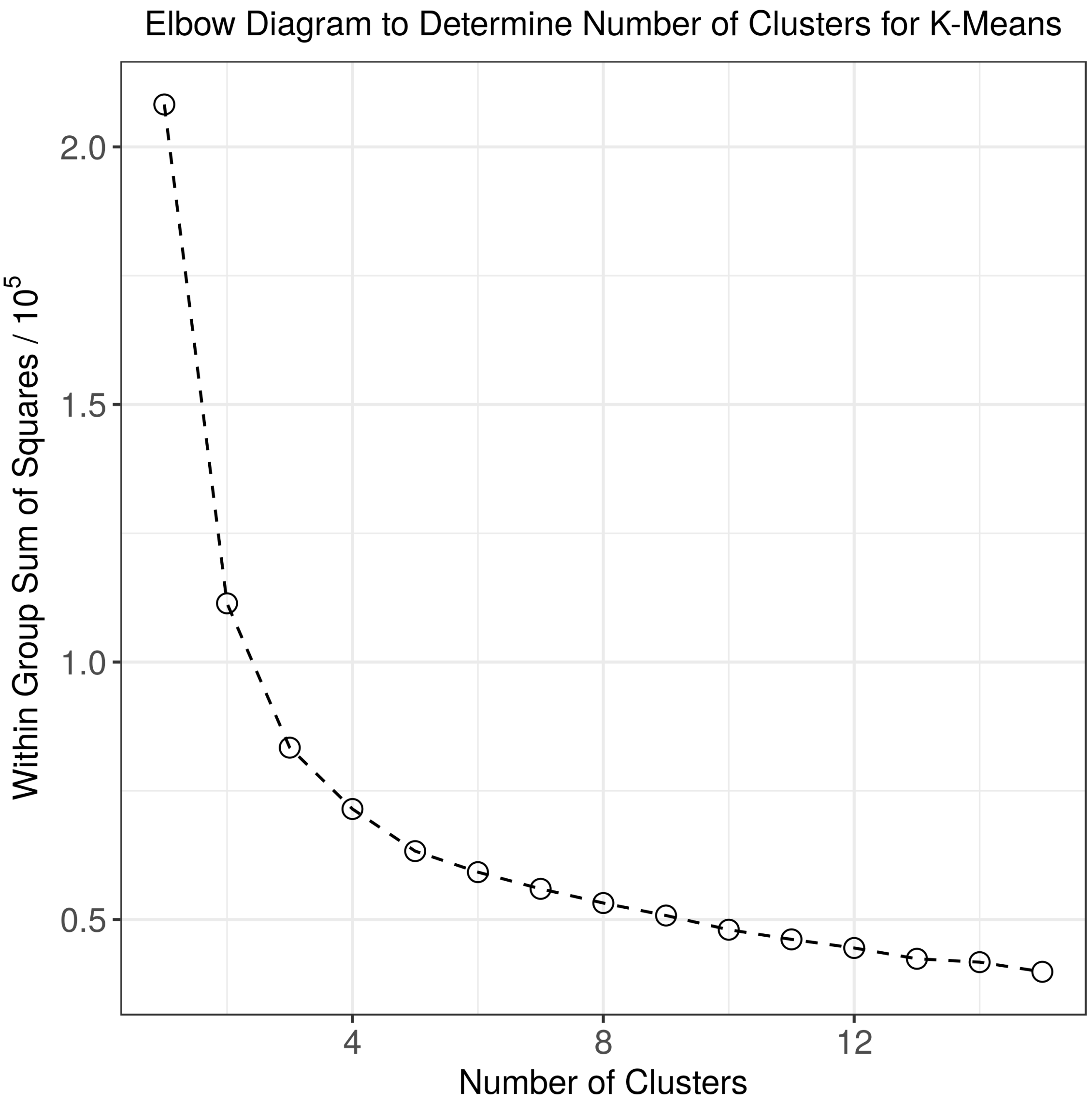

Supplementary Fig. 8: Plot of within-group sum of squares versus the number of k-means clusters. Increasing the number of clusters decreases the within-group sum of squares indicating that cluster members are more similar to each other. However, beyond 4 clusters there is diminishing returns on the within-group sum of squares. This elbow at 4 implies that 4 clusters are appropriate for k-means analysis [10] on our data.

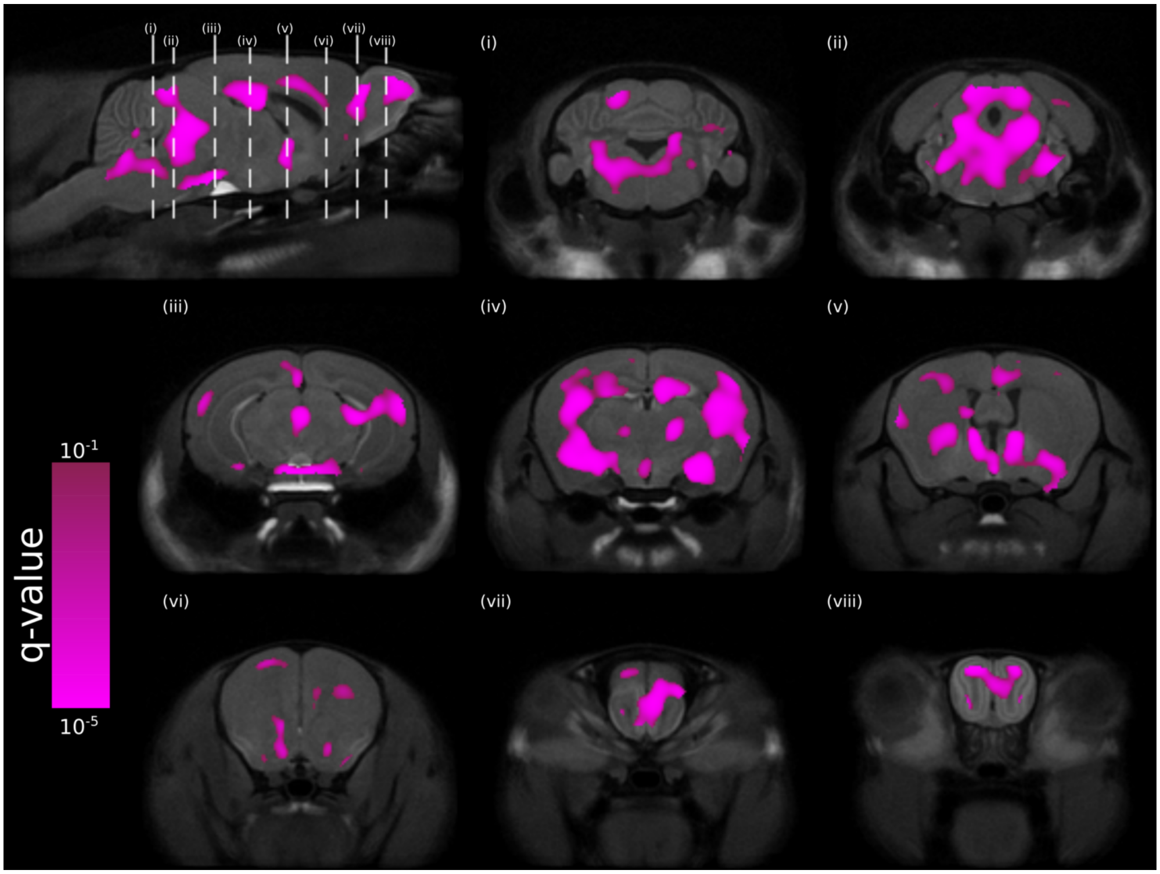

Supplementary Fig. 9: Sexually dimorphic areas in the mouse brain calculated after choosing the p17 age-consensus average as the registration consensus average. Voxelwise statistics were computed similar to those in Figure 4 except that p17 age-consensus average was chosen as the registration consensus instead of p65. The resultant statistics map was in p17 age-consensus space and was transformed to p65 age-consensus space for comparison to Figure 4. A high correlation was observed between these two maps ($r = 0.992$) indicating that choosing p65 as the consensus versus p17 (the median time point) incurs little bias in identifying sexually dimorphic regions.

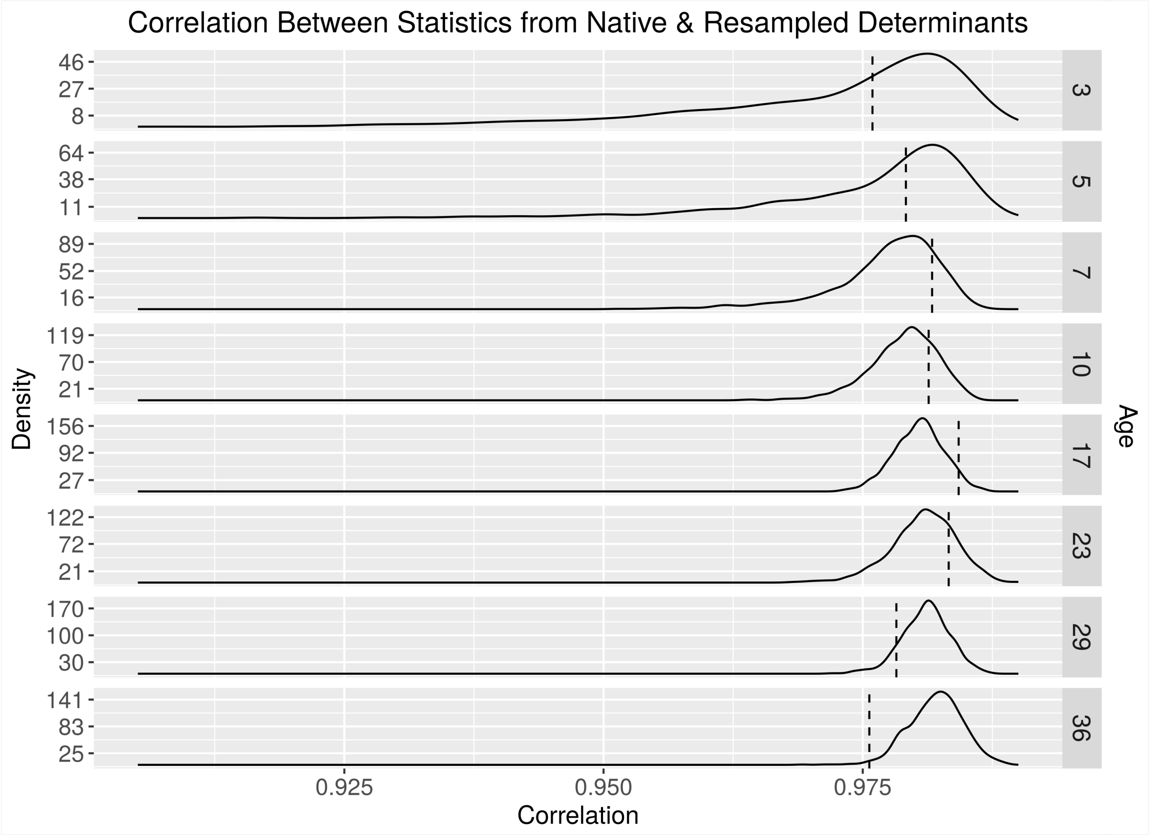

Supplementary Fig. 10: Statistics maps generated without longitudinal registration are similar to those generated with longitudinal registration. Random statistical maps were generated for each time point by permuting sex labels and computing effect size comparing males and females. For every permutation, the effect sizes were calculated for Level 1 determinants (agnostic to longitudinal data and in age-consensus space) and Level 2 determinants (dependent on longitudinal registration and in p65 age-consensus space). The effect sizes from Level 2 determinants were transformed to the age-consensus space corresponding the effect sizes from Level 1 determinants. Correlations are computed between the transformed Level 2 effect size map and Level 1 effect size map and correlation was observed to be high for all time points. The dotted lines indicate correlations when the sex labels are not permuted and correspond to true volumetric effect sizes between males and females.

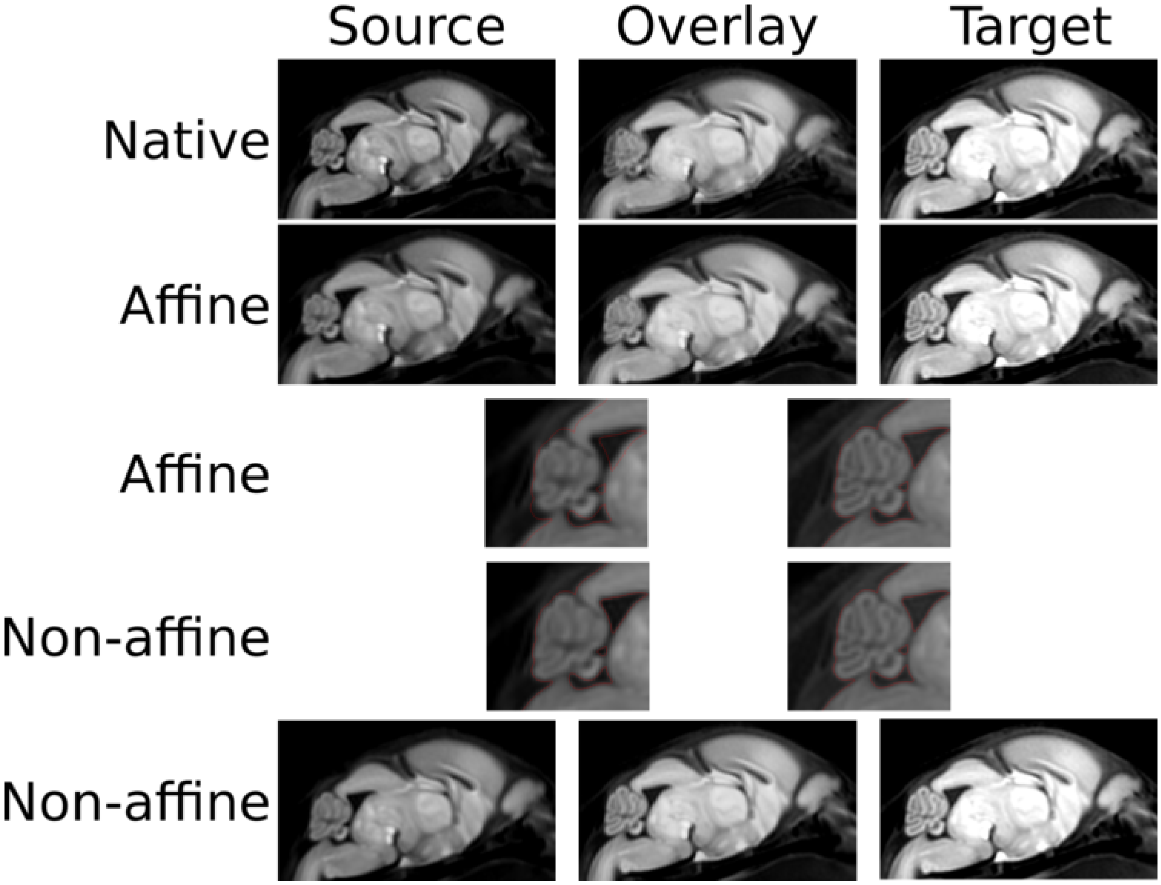

Supplementary Fig. 11: Registration of a source image (p3 average) to a target (p5 average). The native images (after rigid alignment) are on the top row and their overlay is in the middle column. Poor alignment can be found in structures like the cerebellum where there is rapid neonatal growth. Affine registration scales and shears the source image to better align with target image. The affine transformation (generated from the affine registration) is applied to the source image and is shown in the second row. The overlay shows a good match between the affine-transformed source and the target images. However, zooming into the cerebellum of the affine-transformed source and target image (third row) showed that affine registration does not produce proper alignment of the cerebellum. This is illustrated by applying a red contour to the cerebellum of the target image and overlaying this contour to the source image. The non-affine registration corrects this discrepancy (fourth row) and produces the best alignment between source and target images (fifth row).

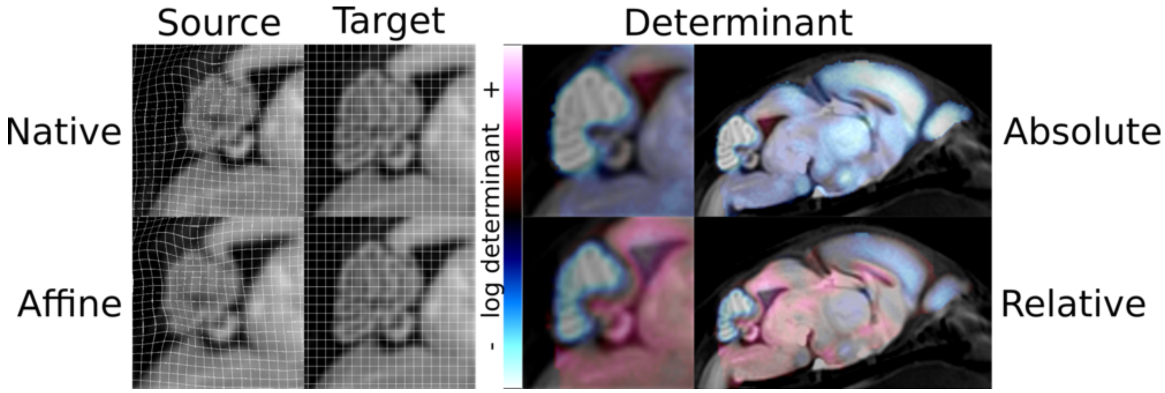

Supplementary Fig. 12: Visualizing deformations caused by transformation of target image using grids and determinants. Illustrated in the left figure, upon transformation of the target image to the source image (this transformation is the inverse of the transformation in Supplementary Figure 11), gridlines in the target image become warped. In the top row, the gridlines warp from transformation to the source image; and in the bottom row, the gridlines warp from transformation to the affine-transformed source. Volumetric changes caused by the transformation can be qualitatively assessed by observing how the volume of a square region (region defined by the open space between gridlines) changes after transformation. It is clear from the convergence of gridlines in the cerebellum that much of the cerebellum decreases in size after transformation. This implies that the cerebellum is smaller in the source image than the target image. Volumetric changes can also be quantified by calculating the determinants (right figure). If a region in the source image is smaller than the corresponding regions in the target image (i.e. gridlines converge), the region has determinants between 0 and 1. Conversely, regions larger in the source image (i.e. gridlines diverge) have determinants larger than 1. Absolute determinants (top row) characterize volumetric changes upon transformation from target to source images and measure the true volumetric differences between target and source images. Relative determinants (bottom row) characterize volumetric changes upon transformation from target to affine-transformed source images and measure the volumetric differences between target and source images upon removal of a scaling factor (this scaling factor makes source and target images the same size as seen in Supplementary Figure 11: second row). The advantage of absolute determinants is that they can be used to calculate the volumes of regions in canonical units like mm^3. Relative determinants, on the other hand, calculate volumes relative to total brain volume instead. However, relative determinants remove whole-brain size variability (which is the largest source of variability among mice [11]) to expose more subtle variations in neuroanatomy.

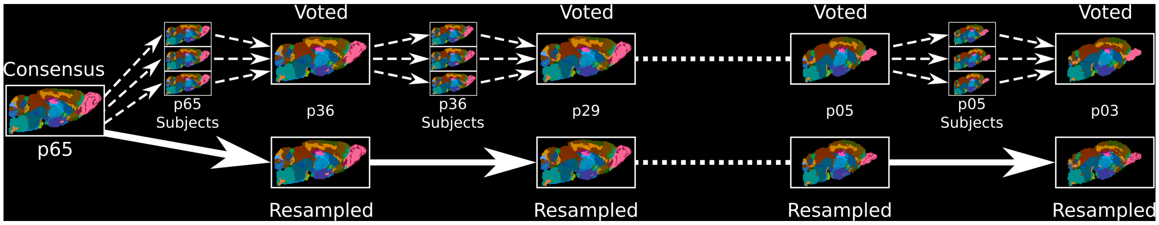

Supplementary Fig. 13: Three different sets of atlases used to check for registration bias in structures. Consensus atlas is the MRI atlas registered to the registration consensus average (which is also the p65 age-consensus average). Since all images are registered to this consensus average, we use this atlas alone to quantify structure volumes in our main study. The two additional sets of atlases created test for different types of biases. The resampled atlases are created by transforming the consensus atlas to every single age in a single interpolation step with transformations obtained from Level 2 of our registrations. This atlas allows us to check for resampling bias as, with this atlas, volumetric information need not be transformed to p65 space prior to quantification of structure volumes. For a given time point, its voted atlas is created by aligning its age-consensus average to the atlas overlaid on every subject of the next immediate-older time point. A voxel voting procedure is taken across all subject atlases to create the voted atlas on the time point's age-consensus average. This method greatly reduces the bias in choosing a single starting adult atlas and transforming it to younger time points. There are multiple intermediate atlases and each time point is only responsible for creating an atlas for the immediate-younger time point.

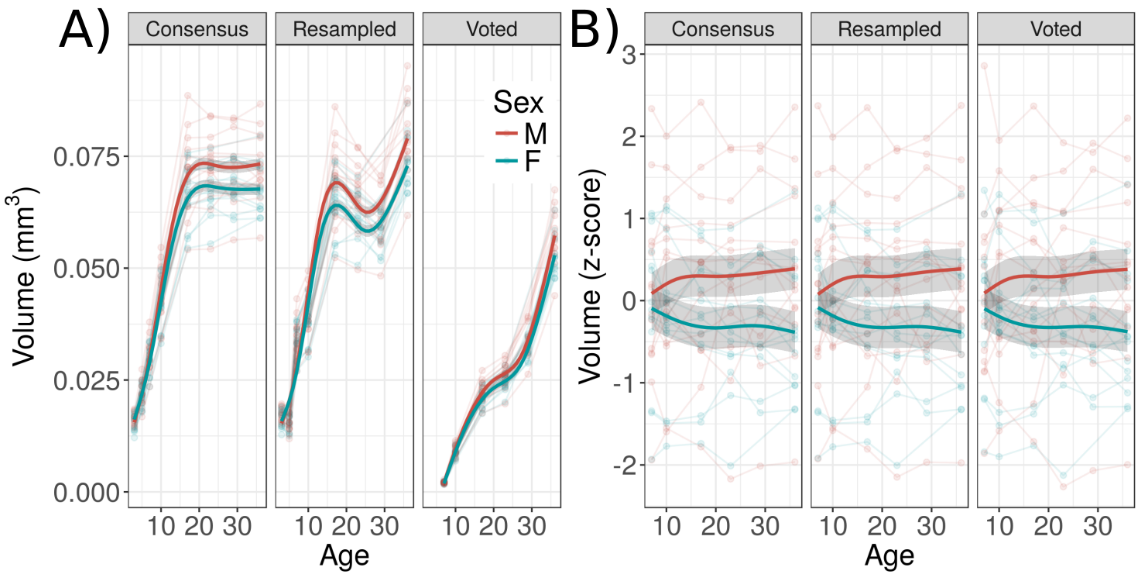

Supplementary Fig. 14: Registration bias exists with atlases but does not discriminate individuals or sex. A) The volume of the Lobule 1-2 white matter estimated using the three sets of atlases: consensus, resampled, and voted (see Supplementary Figure 13). Trendline and standard error (shaded region) were obtained by fitting a linear mixed-effects model. We see that registration bias does play a role in volume estimation of small structures like the Lobule 1-2 white matter as, before p7, voted atlases say this structure does not exist and the other atlases do not agree. B) We computed z-score, removing the overall mean and standardizing variability across the three sets of atlases for each age. We test whether registration bias of structure volumes applies equally across all individuals using two linear mixed-effects models: the first model had z-score volumes as a response variable; fixed effect of time point, sex, and atlas (Consensus, Resampled, or Voted), as well as all interactions; and random effect of individual and individual-atlas interaction. The second model was the same but lacked the random effect of individual-atlas interaction. A log likelihood test showed that registration bias does not discriminate individuals ($\chi^2_5 = 0.33$,P$>$ 0.99). To test if registration bias was the same between the sexes, we performed a similar analysis, except the second model lacked all interactions between sex and atlas. We found that registration bias does not discriminate sex ($\chi^2_{12} = 0.08$,P$>$ 0.99). All other structures showed little effects of registration bias affecting each individual differently (uncorrected all P>0.93) or affecting each sex differently (uncorrected all P>0.999). This supports our conclusion that while registration bias may exist in structure volume measurements, this bias applies equally to all individuals and sexes.

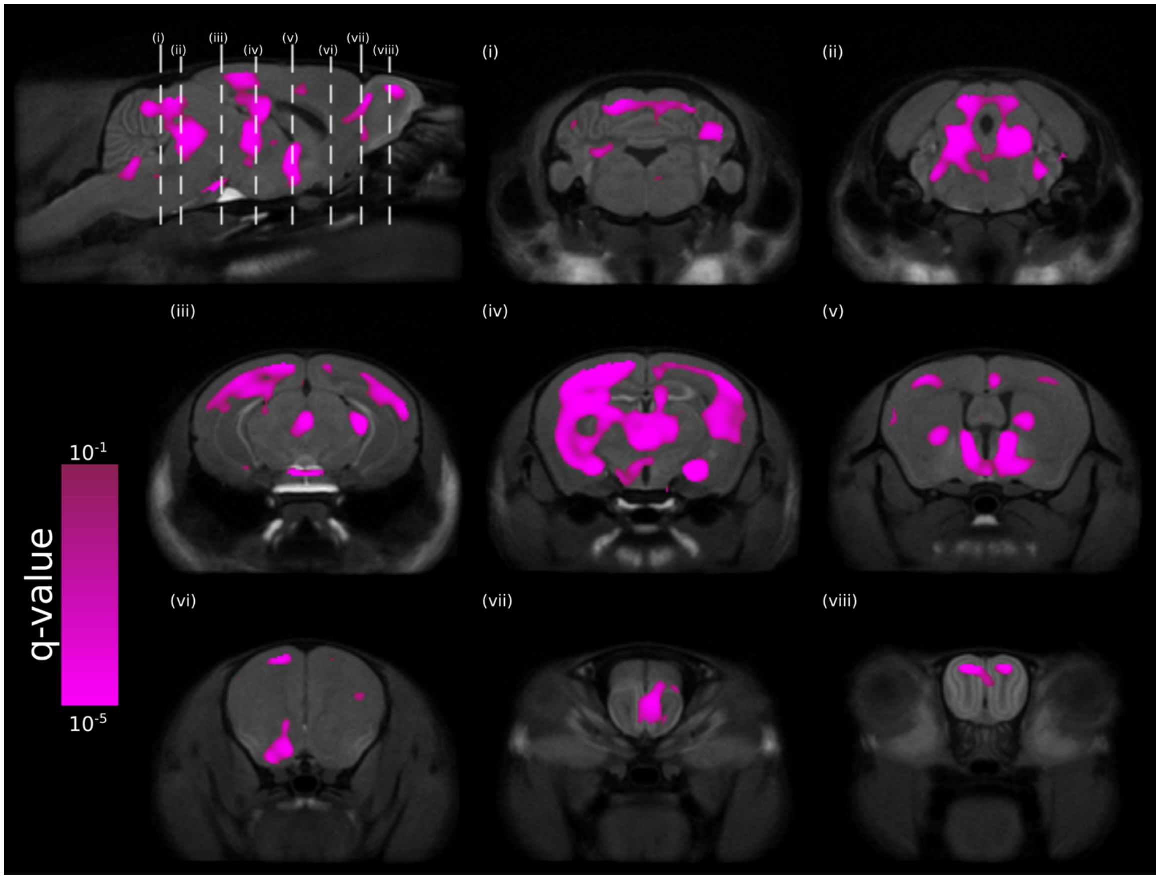

Supplementary Fig. 15: Sexually dimorphic voxels when using time point instead of age as a predictor. Our time points are not evenly spaced in development with more time points concentrated in early life. We assessed whether this has an effect on the sexually dimorphic regions identified by using time point as a categorical predictor. We found more sexually dimorphic voxels compared to Figure 4, but many of the same regions are implicated in both figures.

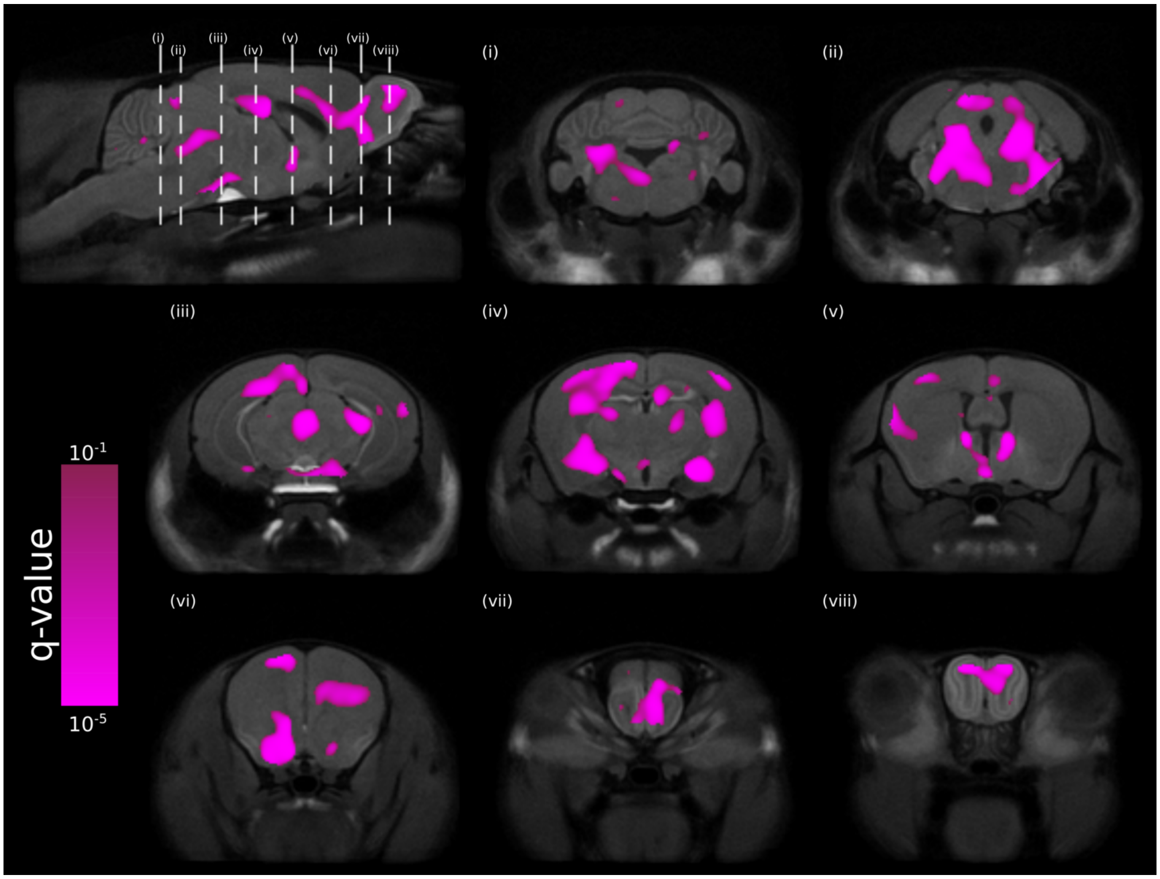

Supplementary Fig. 16: Sexually dimorphic voxels in the mouse brain after removal of all data corresponding to p65. The figure was generated in a similar manner to Figure 4, except all data from p65 was removed prior to statistical analysis. This was done as p65 is almost a month after the previous p36 time point and we wanted to assess whether this type of sampling would have any effect. Since the results are similar to Figure 4, we conclude that this does not for our study.

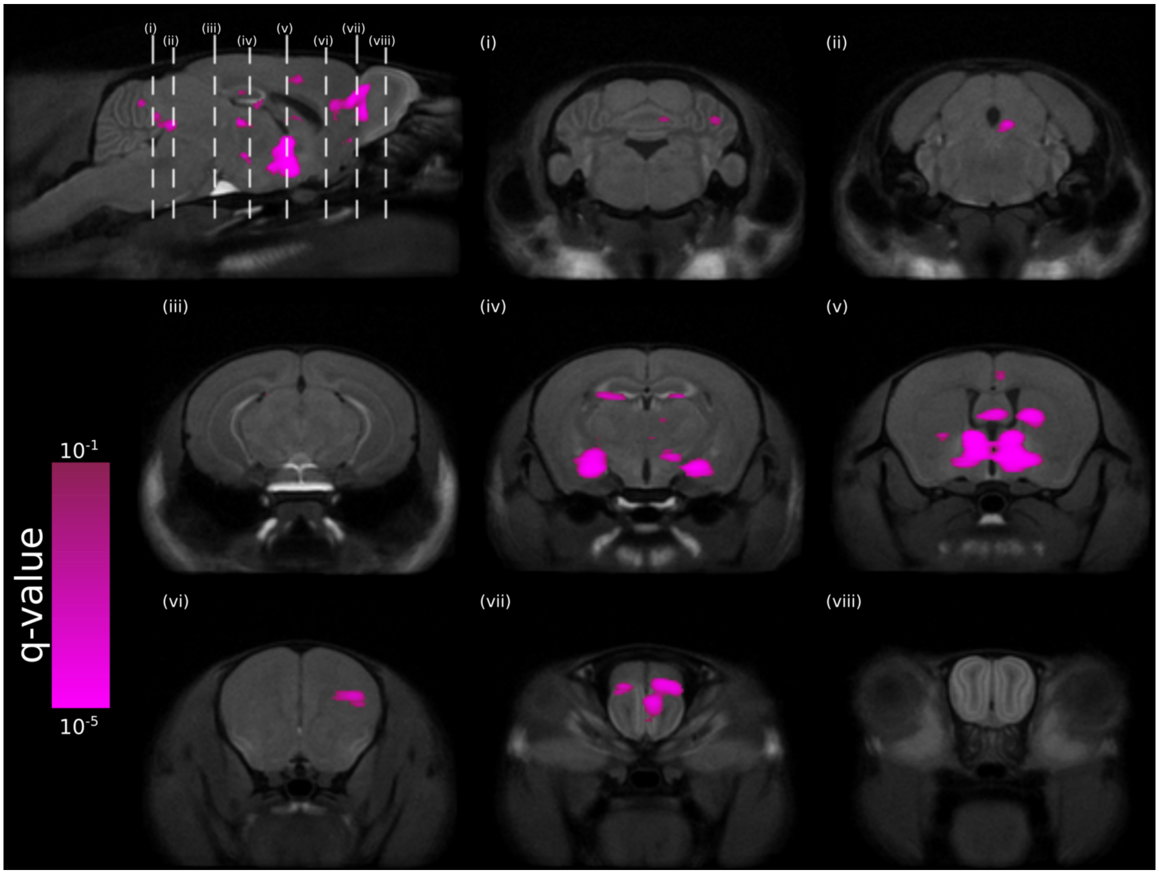

Supplementary Fig. 17: Voxels with sexually dimorphic absolute Jacobian determinants. The analysis was similar to Figure 4 except absolute Jacobian determinants were used as the dependent variable instead of relative determinants. In addition, 6th-order natural splines were used to model fixed effect of age and 2nd-order natural splines were used to model random effect of age. We observed the absolute volumes capture sexual dimorphisms in similar regions of the brain as relative volumes; however, relative volumes capture dimorphisms larger in females in regions like the somatosensory cortex, midbrain, and pons.

Supplementary References

1. Collins, D. L., Neelin, P., Peters, T. M. & Evans, A. C. Automatic 3D intersubject registration of MR volumetric data in standardized Talairach space. *Journal of computer assisted tomography* **18,** 192–205 (1994).
2. Avants, B. B., Tustison, N. & Song, G. Advanced Normalization Tools (ANTS). *Insight J* (2009).
3. Chakravarty, M. M. *et al.* Performing label-fusion-based segmentation using multiple automatically generated templates. *Human brain mapping* **34,** 2635–2654 (2013).
4. Lein, E. S. *et al.* Genome-wide atlas of gene expression in the adult mouse brain. *Nature* **445,** 168–176 (2007).
5. Thompson, C. L. *et al.* A high-resolution spatiotemporal atlas of gene expression of the developing mouse brain. *Neuron* **83,** 309–323 (2014).
6. Morey, R. D. & Rouder, J. N. *BayesFactor: Computation of Bayes Factors for Common Designs* R package version 0.9.12-2 (2015). <`https://CRAN.R-project.org/package=BayesFactor`>.
7. Schwarz, G. *et al.* Estimating the dimension of a model. *The annals of statistics* **6,** 461–464 (1978).
8. Liaw, A. & Wiener, M. Classification and Regression by randomForest. *R News* **2,** 18–22 (2002).
9. Reuter, M., Schmansky, N. J., Rosas, H. D. & Fischl, B. Within-subject template estimation for unbiased longitudinal image analysis. *Neuroimage* **61,** 1402–1418 (2012).
10. Tibshirani, R., Walther, G. & Hastie, T. Estimating the number of clusters in a data set via the gap statistic. *Journal of the Royal Statistical Society: Series B (Statistical Methodology)* **63,** 411–423 (2001).
11. Lerch, J. P. *et al.* Wanted dead or alive? The tradeoff between in-vivo versus ex-vivo MR brain imaging in the mouse. *Frontiers in neuroinformatics* **6,** 6 (2012).
