# Supplementary material for: Mouse MRI shows brain areas relatively larger in males emerge before those larger in females
Source: Nat Commun. 2018 Jul 5;9:2615. doi: 10.1038/s41467-018-04921-2 (PMC6033927; doi:10.1038/s41467-018-04921-2)
Supplement: Supplementary file 3 — Description of Additional Supplementary Files [file 41467_2018_4921_MOESM3_ESM.pdf]

Description of Additional Supplementary Files

Supplementary Movie 1: Animation of mouse brain development. The average MR image at every experimental time point were sequentially registered together. These registrations create transformations in the form of vector fields that map the average of each age time point to the average of the immediate-older age time point. To create an animation of the growing brain, piecewise quadratic polynomials were used to interpolate between the experimental time points. At every voxel, 4 independent quadratic polynomials were needed - one for each of the 3 components of the vector field denoting transformations and one for the scalar field denoting image intensity. The interpolated data was then used to create an animation of a sagittal slice of the mouse brain.

Supplementary Movie 2: Animation of sexual dimorphisms in a coronal slice showing the BNST and MPON. Animation was created using a similar procedure to Supplementary Movie 1, with the exception that an additional quadratic polynomial was used at every voxel to interpolate the *t*-statistics for the main effect of sex from age-centered linear-mixed effects models. Colour scale bar for the *t*-statistics are shown in Figure 5.

Supplementary Movie 3: Animation of sexual dimorphisms in a coronal slice showing the MeA. Animation was created using a similar procedure to Supplementary Movie 2. Colour scale bar for the *t*-statistics are shown in Figure 5.

Supplementary Movie 4: Animation of sexual dimorphisms in a coronal slice showing the PAG. Animation was created using a similar procedure to Supplementary Movie 2. Colour scale bar for the *t*-statistics are shown in Figure 5.

Supplementary Data 1: Full list of genes from the Allen Brain Institute, and their preferential spatial expression in sexually dimorphic regions.
